# A Review of the Relationship Between Vitamin D and Parkinson Disease Symptoms

**DOI:** 10.3389/fneur.2020.00454

**Published:** 2020-05-27

**Authors:** Michelle E. Fullard, John E. Duda

**Affiliations:** ^1^Department of Neurology, University of Colorado, Denver, CO, United States; ^2^Parkinson's Disease Research, Education and Clinical Center, Corporal Michael J. Crescenz VA Medical Center, Philadelphia, PA, United States; ^3^Department of Neurology, University of Pennsylvania School of Medicine, Philadelphia, PA, United States

**Keywords:** vitamin D, Parkinson's disease, motor symptoms, review, non-motor symptoms

## Abstract

Vitamin D is a fat-soluble secosteroid that exerts its effects by binding to the vitamin D receptor (VDR), through which it directly and indirectly modulates the expression of hundreds to thousands of genes. While originally known for its role in regulating calcium homeostasis and metabolism, vitamin D is now associated with many other health conditions, including Parkinson's disease (PD). A high prevalence of vitamin D deficiency has been noted in PD for at least the past two decades. These findings, along with the discovery that the VDR and 1α-hydroxylase, the enzyme that converts vitamin D to its active form, are highly expressed in the substantia nigra, led to the hypothesis that inadequate levels of circulating vitamin D may lead to dysfunction or cell death within the substantia nigra. Studies investigating the relationship between vitamin D status and PD, however, have been inconsistent. Two prospective studies examined the association between mid-life vitamin D levels and risk of PD and produced conflicting results—one demonstrated an increased risk for PD with lower mid-life vitamin D levels, and the other showed no association between vitamin D and PD risk. One of the most consistent findings in the literature is the inverse association between serum vitamin D level and motor symptom severity in cross-sectional studies. While these data suggest that vitamin D may modify the disease, another likely explanation is confounding due to limited mobility. Fall risk has been associated with vitamin D in PD, but more study is needed to determine if supplementation decreases falls, which has been demonstrated in the general population. The association between vitamin D and non-motor symptoms is less clear. There is some evidence that vitamin D is associated with verbal fluency and verbal memory in PD. Studies in PD have also shown associations between vitamin D status and mood, orthostatic hypotension and olfactory impairment in PD. While more research is needed, given the numerous potential benefits and limited risks, vitamin D level assessment in PD patients and supplementation for those with deficiency and insufficiency seems justified.

## Introduction

Vitamin D refers to a group of fat-soluble secosteroids that can be obtained through dermal synthesis by exposure to sunlight or though dietary intake (1). Vitamin D itself is biologically inert and must undergo two hydroxylations to become active. The liver converts vitamin D to 25-hydroxyvitamin D and then the kidney and other tissues convert 25-hydroxyvitamin D to the active form 1,25-dihydroxyvitamin D, also known as calcitriol (1). Vitamin D exerts its effects by binding to the vitamin D receptor (VDR), through which it directly and indirectly modulates the expression of hundreds to thousands of genes (1, 2). While originally known for its role in regulating calcium homeostasis and metabolism, vitamin D was reported in the 1930s and 1940s to improve conditions like psoriasis, asthma and rheumatoid arthritis (3, 4). It is now associated with many other health conditions, such as cardiovascular disease, cancer, autoimmune disease and neurodegenerative disease, including Parkinson's disease (PD) (5).

Multiple mechanisms have been proposed for the role of vitamin D in neurodegenerative diseases. Vitamin D may confer neuroprotection through the action of neurotrophic factors, regulation of nerve growth or through protection against cytotoxicity (6). In several studies, the synthesis of neurotrophic factors, including neurotrophin 3 (NT3) and glial cell line-derived neurotrophic factor (GDF), were upregulated by 1,25(OH)_2_D3 (7–12). Riaz et al. and Wang et al. showed that vitamin D induced stimulation of neurotrophins was correlated with a neuroprotective effect in rat models (13, 14). Through the VDR, Vitamin D can upregulate nerve growth factor (NGF) (7, 15), and in a rat model of peripheral nerve injury, rats exposed to vitamin D2 demonstrated significantly increased axogenesis and axon diameters (16).

Vitamin D may also modulate the toxicity of reactive oxygen species. Nitric oxide is produced by inducible nitric oxide synthase (iNOS), an enzyme that is induced in CNS neurons and non-neuronal cells as part of an immune response (17). At high levels, nitric oxide can damage neurons, and vitamin D has been shown to inhibit the synthesis of iNOS, thereby reducing levels of nitric oxide (17, 18). In hippocampal neurons, the presence of vitamin D was associated with downregulation of L-type voltage-sensitive Ca^2+^ channel and was correlated with a neuroprotective effect at physiologic doses (19). Lastly, vitamin D may exert a neuroprotective effect through reducing oxidative stress. In a study in which dopaminergic and non-dopaminergic neurons were exposed to glutamate, pretreatment with vitamin D protected both cell types against cytotoxicity in a dose-dependent manner (20).

The high prevalence of vitamin D deficiency in Parkinson's disease was first noted by Sato and colleagues in 1997 (21). Since then, multiple studies have demonstrated a higher prevalence of vitamin D deficiency and insufficiency in PD compared to controls (22–24). After the discovery that the VDR and 1α-hydroxylase, the enzyme that converts vitamin D to its active form, were highly expressed in the substantia nigra, it was hypothesized that inadequate levels of circulating vitamin D may lead to dysfunction or cell death within the substantia nigra (25, 26). Adding further support to this hypothesis were VDR knockout mice that demonstrated motor impairment and reduced stride length (27), and animal and cell culture studies that demonstrated neuroprotective effects of vitamin D against MPTP and 6-OHDA exposure (28–30). Over the last two decades, multiple studies have examined the relationship between low levels of vitamin D and Parkinson's disease. In this manuscript, we will review the evidence for the effect of hypovitaminosis D on Parkinson's disease risk, symptoms and progression.

## Parkinson's Disease Risk

Over the past decade, several studies have attempted to determine the relationship between hypovitaminosis D and the risk of Parkinson's disease, with conflicting results (Table 1). The first study to examine the association between vitamin D levels in mid-life and the risk of Parkinson's disease was performed using a nationwide drug-reimbursement database in Finland (31). Of the 3,173 men and women included in the study, 50 went on to develop Parkinson's disease over the 29-year follow-up period. After adjusting for potential confounders, participants in the highest quartile for baseline serum vitamin D level had a 65% lower risk of developing PD than those in the lowest quartile, suggesting that lower levels of vitamin D in mid-life may increase the risk of Parkinson's disease (31). In a case control study in elderly Chinese, self-reported outdoor activity and total vitamin D intake were inversely associated with Parkinson's disease, suggesting vitamin D may be protective against the development of PD (32). However, recall bias and the lack of serum vitamin D levels make it difficult to draw firm conclusions from this study. In addition, outdoor physical activity was used as a proxy for vitamin D status, but there is compelling data that physical activity reduces the risk of Parkinson's disease, which is a likely confounder in this study (33).

**Table 1:** Association of Vitamin D with Parkinson's disease risk and symptoms.

	**Study findings**	**References**
**Parkinson's Disease Risk**		
Vitamin D and Parkinson's Disease Risk	Findings are mixed. A study in Finland found an association between vitamin D level and future PD risk while a study in the United States did not. Differences in the populations studied may play a role	(31–36)
Vitamin D Receptor and Parkinson's Disease Risk	The SNP FokI is associated with a decreased risk of PD in Asian populations but not in Caucasian populations	(37–40)
**Motor Symptoms**		
Disease Severity	Lower serum vitamin D levels are consistently associated with higher motor symptom severity in PD, however, reduced mobility in advanced disease may result in limited sun exposure and lower vitamin D levels	(22, 41–47)
Disease Progression	Findings are mixed. In two studies, lower baseline vitamin D level was a predictor of increased motor severity, while two other studies found no association. There is risk for confounding due to limited mobility and sun exposure as the disease progresses	(22, 48–53)
Balance and Falls	Lower vitamin D levels are associated with falls. High dose vitamin D supplementation may improve balance in younger PD patients. More recent data in the general population suggests there is a U-shaped response curve to vitamin D supplementation	(45, 54–75)
**Non-motor Symptoms**		
Cognition	In PD without dementia, higher plasma vitamin D levels are associated with better verbal fluency and verbal memory. The VDR FokI AA genotype is associated with faster cognitive decline and lower VDR activity	(44, 50, 76, 77)
Mood	There is limited evidence of an association between depression and lower serum vitamin D levels in PD. Data is inconsistent in the general population, however, a meta-analysis of 31,000 participants found lower vitamin D levels in participants with depression compared to controls	(44, 73, 78–84)
Orthostatic hypotension	Lower serum vitamin D levels were associated with orthostatic hypotension in one study involving PD patients and multiple studies involving the general population, however, the findings have been inconsistent	(85–90)
Olfactory function	One study in PD reported a negative correlation between odor identification scores and serum vitamin D level. More studies are needed	(91)

*UPDRS, Unified Parkinson's Disease Rating Scale; H&Y, Hohn and Yahr; SNP, Single Nucleotide Polymorphism*.

More recent data have failed to demonstrate an association between vitamin D levels and Parkinson's disease risk. In contrast to the study in Finland, an analysis from the prospective Atherosclerosis Risk in Communities (ARIC) study cohort of almost 13,000 men and women in the United States found no association between baseline vitamin D level and risk of PD 17 years later (34). In a smaller study of participants at high risk for Parkinson's disease in the Parkinson Associated Risk Syndrome (PARS) study, there was no difference in baseline serum vitamin D level between the high risk group (dopamine transporter scan deficit and hyposmia) vs. the lower risk group (35). Taken together, these more recent data do not support the hypothesis that chronic vitamin D insufficiency contributes to PD pathogenesis. All of these studies, however, were based on a single serum measurement of vitamin D. To try to overcome some of these limitations, Larsson et al. performed a mendelian randomization study to determine if genetically decreased vitamin D concentrations are associated with PD (36). They used 4 single-nucleotide polymorphisms (SNPs) associated with serum vitamin D level and found no association between these genetic variants and PD (36). The authors noted that these four SNPS only explain about 3.6% of the variation in 25-hydroxyvitamin D concentrations, which would limit the power to detect a weaker association between these SNPs and PD (36).

Potential explanations for the inconsistent findings between the two main prospective studies include differences in geography, behaviors, and economics of the populations studied (92). Vitamin D levels in the Finnish population were much lower than in the US population, so it is possible vitamin D levels below a certain threshold are required to increase the risk of PD. In addition, diet and activity levels may differ between the populations, both of which are associated with vitamin D levels and PD risk (93). Given the differences in geography, environmental risk factors for PD may differ as well.

Multiple meta-analyses have examined the association between VDR polymorphisms and risk of Parkinson's disease (37–40). The most recent and comprehensive meta-analysis by Wang et al. included the SNPs FokI, TaqI, ApaI, and Bsm and included 5–7 studies per SNP with over one thousand PD patients and controls (37). They found that FokI was significantly associated with decreased risk of PD in most models (allelic, dominant, and additive) but not in a recessive model. However, when stratified by race, FokI was associated with PD susceptibility in all four models in Asian populations while there was no significant association in any of the models in the Caucasian population. TaqI was associated with an increased risk of PD in a dominant model only, and there were no significant associations between ApaI and Bsm in any model. In addition, on sensitivity analysis, omission of any one of five studies for TaqI resulted in a lack of association (37). More work is needed in this area with larger numbers and more diverse populations.

## Motor Symptoms

### Disease Severity

The motor symptoms of Parkinson's disease include bradykinesia, rigidity, rest tremor and postural instability. Symptom severity can be measured using several scales, including the Unified Parkinson's Disease Rating Scale (UPDRS) (41), as well as the Hoehn and Yahr stage (H&Y) (42). One of the most consistent findings in the literature is the association between serum vitamin D levels and motor symptom severity in PD. In multiple cross-sectional studies, vitamin D level was correlated with the motor portion of the UPDRS (22, 43–47), and H&Y stage (43). Suzuki et al. found a significant, inverse relationship between serum vitamin D level and part III of the UPDRS in 137 PD participants (43). Similarly, vitamin D level was highest in participants with H&Y stage 1–1.5 (mean 23.9 ng/ml) and lowest in participants who were H&Y stages 4–5 (mean 14.5 ng/ml) and demonstrated a dose-response relationship (43). A second study using data from 388 PD patients in the Harvard Biomarker Study had similar findings. Total UPDRS score was inversely correlated with serum vitamin D level (*p* = 0.02) after adjusting for age, sex, race and vitamin D supplementation (22). In this study, however, there was no association between vitamin D level and H&Y stage.

While these data, along with preclinical studies in mice and cell culture (28–30), suggest that vitamin D modifies the disease, another very plausible explanation is confounding due to mobility. Increasing disease severity is associated with a decline in mobility, which leads to decreased sun exposure and decreased dermal synthesis of vitamin D. Therefore, the lower vitamin D levels documented in more advanced PD may be a result of limited sun exposure and represent reverse causation, rather than an effect of lower vitamin D levels on PD severity.

Associating genotype and clinical outcome is one way to try to avoid reverse causality. The evidence involving vitamin D receptor (VDR) polymorphisms and Parkinson's disease risk and severity, however, has been inconsistent (37). In a study by Suzuki, the FokI CC genotype for VDR polymorphism was associated with a milder form of PD, but vitamin D level was not (43). In an intervention study performed by the same group, 114 PD patients were randomized to receive either 1,200 units of vitamin D3 daily or placebo for 12 months (94). The H&Y stage was stable in the treatment group, but significantly declined in the placebo group. FokI genotype modified the relationship between vitamin D supplementation and motor progression. For participants with the FokI CC genotype, vitamin D supplementation did not affect progression (compared to the placebo group), but Vitamin D supplementation prevented deterioration in those with FokI TT (94). This genotype has been shown to have less transcriptional activity (76), and therefore individuals with this genotype may require higher serum levels of vitamin D to perform the same functions as those with the CC genotype. More study in larger populations is needed to determine if this is a stable finding.

### Progression

Four studies have evaluated the relationship between disease progression and serum vitamin D level in Parkinson's disease and one study has evaluated the relationship between vitamin D supplementation and disease progression (Table 1). Evatt et al. used data from 157 participants in the DATATOP study to evaluate the prevalence of vitamin D insufficiency in early, untreated PD, as well as examine the association between vitamin D levels and disease progression (48). There were high rates of vitamin D deficiency and insufficiency in the PD participants, but vitamin D level unexpectedly increased by a small amount over the course of the study (median follow-up of 13 months). There was no relationship between vitamin D level and disease progression (48). Similarly, 624 PD patients from Oxford's Parkinson's Disease Centre Discovery cohort had baseline serum vitamin D measured and were followed for a mean of 3.2 years, and no association was found between baseline vitamin D level and disease progression measured by the MDS-UPDRS III (49). However, reduced levels of vitamin D were associated with worse activities of daily living at baseline as measured by MDS-UPDRS II. In a third study, 145 newly diagnosed Parkinson's patients and 94 age-matched controls in the North East of England had serum vitamin D levels checked at baseline and 18 months (50). The vitamin D levels at these two time points were significantly lower in the PD participants compared to controls. Lower baseline vitamin D level was a predictor of increased motor severity at 36 months as measured by the MDS-UPDRS III (50). Lastly, Ding and colleagues found that lower vitamin D levels at baseline predicted a greater change in UPDRS score at follow-up (12–25 months) after adjusting for age, sex, race and vitamin D supplementation (22). Similar to the cross-sectional studies demonstrating an association between serum vitamin D level and motor severity, these studies on motor progression are also at risk for confounding due to more limited mobility as the disease progresses.

Using data from the National Institutes of Health Exploratory Trials in Parkinson's Disease (NET-PD) Long-term study, Luthra et al. examined disease progression at 3 years in patients with early PD who took 400 IU or more per day of vitamin D compared to those who did not take supplements (51). Of the 1,741 participants, only 12% were taking a vitamin D supplement and 34% were taking a multivitamin that contained vitamin D. At 3 year follow-up, there was no difference in UPDRS scores between those taking vitamin D and those who were not.

Data regarding the effects of vitamin D supplementation on Parkinson's disease progression is sparse. A case report from 1997 described a 50 year old patient with 13 years of PD symptoms somewhat responsive to levodopa therapy, bone abnormalities, low serum calcium and phosphorus and low serum vitamin D (52). Vitamin D3 supplementation at 4,000 IU daily of 25-hydroxyvitamin D3, roughly equivalent to 20,000 IU of vitamin D3 (53), and calcium supplements were added to his regimen and his lab values normalized. In addition, his parkinsonism significantly improved over the following year and he was able to reduce his levodopa by half (52). While this is an extreme case, it highlights the need for more study on the effects of vitamin D supplementation on PD symptoms. To date, only two small randomized, placebo-controlled trials have been done examining the effects of vitamin D supplementation on motor symptoms and progression in PD. As discussed above, Suzuki et al. randomized 114 PD patients to receive 1,200 IU/day of vitamin D3 or placebo for 12 months. The serum vitamin D level doubled for those in the intervention group and remained unchanged for the placebo group. H&Y stage was stable in the intervention group, while H&Y stage significantly worsened for the placebo group (+0.33) (94). In a second study in Iran, 120 PD patients with levodopa-induced dyskinesia were randomized to receive either 1,000 IU of vitamin D per day or placebo (95). At 3 month follow-up, there was no difference in levodopa-induced dyskinesia as measured by the UPDRS IV sub score and no difference in the UPDRS motor score, suggesting that vitamin D has no effect on dyskinesia. The follow-up time, however, was too short to determine if vitamin D supplementation influenced motor progression and the dose of vitamin D supplementation was low.

Both of these studies used low doses of vitamin D supplementation, which may have limited the potential to detect improvement in symptoms. A report in 2005 noted that 10–15 min of whole-body exposure to sunlight in the peak of summer can generate 10,000–25,000 IU of vitamin D3, concluding that the recommendations for vitamin D intake of 200–800 IU/day are likely very inadequate (96, 97). A study from 1977 showed that a daily intake of 10,000 IU daily resulted in serum vitamin D levels most similar to the subjects who were exposed to daily whole-body artificial ultraviolet light (53). One of the reasons often cited for giving lower doses of vitamin D supplementation is the concern for toxicity and hypercalcemia, however, multiple studies have shown that toxicity is rare and high serum levels of vitamin D are not strongly correlated with hypercalcemia (4, 98). When hypercalcemia occurs, it is often easily reversed (4, 98) One state psychiatric hospital in Cincinnati, Ohio offered all 4,700 patients admitted to the facility from 2011 to 2018 supplements of either 5,000 IU/d or 10,000 IU/d of vitamin D3, many of whom were on the supplements for over 12 months (4). Serum 25(OH) D levels were frequently above 100 ng/ml for patients taking doses of 10,000 IU/d, with a range up to 202 ng/ml. None of these patients developed hypercalcemia, nephrolithiasis or other adverse effects and the authors concluded that long-term supplementation at doses of 5,000 to 10,000 U/d appear safe (4). Given the large range of vitamin D supplementation that has been reported in the literature, and the low doses of supplementation provided in the two PD RCTs, more high quality studies with a range of doses are needed to determine if vitamin D is effective for PD motor symptoms and progression and at what doses.

### Balance and Falls

Falls are a major determinant of poor quality of life, morbidity and mortality in Parkinson's disease (99, 100). Balance problems and postural instability are the leading cause of falls, with up to 70% of PD patients experiencing at least one fall per year (101–105). Over the past two decades, there has been increasing evidence that vitamin D supplementation can improve falls in older adults, which prompted two studies in the Parkinson's population that evaluated the relationship between vitamin D and balance and falls.

Most of what is known about the association between vitamin D and falls comes from literature in the older, non-PD population. There is a high prevalence of falls among adults ages 65 years and older, which, similar to PD, can lead to fractures and increased morbidity, mortality and cost (106–108). The rate of vitamin D insufficiency is higher in fallers, estimated up to 70%, compared to 40–50% in non-fallers over the age of 65 (54–56). One of the main mechanisms of falls in the elderly is reported to be impairment of postural reactions—either altered or delayed—due to abnormalities in musculoskeletal mechanisms or processing of sensorimotor information (57–59). Low serum vitamin D levels have been associated with increased postural sway (60) and balance (61) and vitamin D supplementation was shown to improve balance and body sway (62) and stabilize postural equilibrium in the elderly (63). In addition, multiple RCTs have shown a decrease in fall rate and fracture rate with vitamin D supplementation in older adults (64–67), especially in those who were vitamin D insufficient at baseline and received at least 800 IU per day (68). In a meta-analysis of five randomized controlled trials of vitamin D supplementation including 1,237 participants, vitamin D supplementation reduced the odds ratio of falling by 22% compared to patients receiving calcium or placebo and further reduced falls in older women by 46–65% (64).

More recent data, however, has suggested that there is a U-shaped response curve when it comes to fall reduction secondary to vitamin D supplementation (69–72). In one study, there was no decrease in falls on low dose vitamin D (400–800 IU), significant decrease in falls on medium doses (1,600–3,200 IU) and no decrease on high doses (4,000 IU) (72). Similarly, when the 12 month serum 25(OH)D levels were divided into quintiles, fall rates also followed a U-shaped curve. The greatest decrease in falls occurred in the group with a final serum 25(OH)D level of 32–38 ng/ml and fall rates increased when the serum 25(OH)D level exceeded 40–45 ng/ml (72). Further trials are needed to determine the appropriate dose of vitamin D supplementation and target serum vitamin D level to reduce falls.

Patients with Parkinson's disease are more likely to fall than their age-matched peers and falls in this population can have devastating consequences (109). While falls have been associated with vitamin D level in Parkinson's disease (73), few studies have examined the mechanisms related to these falls, and only one RCT has examined the effects of vitamin D supplementation on balance. In a study of forty PD patients with a mean H&Y stage of 2.6, serum vitamin D levels were correlated with symmetry of automatic postural responses measured by the Motor Control Test (MCT), which assesses the subject's ability to recover after an unexpected external disturbance (74). In the PD participants, lower vitamin D concentration was correlated with a higher degree of asymmetry in the postural response after adjusting for motor severity (74). In this study, the authors also measured postural sway and found that postural sway was correlated with vitamin D serum levels in only one out of six conditions (45). To determine if vitamin D supplementation improved balance, similar to non-PD older adults, a pilot RCT was performed which involved 16 weeks of high dose vitamin D (10,000 IU/day) or placebo in 51 PD subjects. The authors found that high dose supplementation was well-tolerated and resulted in a doubling of serum concentrations (75). There was no improvement in the primary endpoint of balance as measured by the Sensory Organization Test (SOT). However, a *post hoc* analysis found that younger participants (ages 52–66 years) had significant improvement in the SOT at 16 weeks compared to the older participants (ages 67–86 years), suggesting a potential role for high dose vitamin D supplementation in younger PD patients (75). Given the more recent data consisting of increased falls with high doses of vitamin D in the non-PD population, more studies with longer follow-up are needed to determine an appropriate dose of vitamin D for PD patients at risk for falls.

## Non-Motor Symptoms

### Cognition

While many studies have demonstrated an association between vitamin D level and cognition in the general population, the few clinical trials that have been done fail to show improvement with supplementation (110–119). Even less is known about the effects of vitamin D on cognition in Parkinson's disease, and study findings have been inconsistent (Table 1).

In a study by Peterson el al, 286 participants with PD underwent neuropsychiatric testing and serum vitamin D testing at baseline (44). Models were adjusted for age, disease duration and Hoehn and Yahr stage. In the non-demented cohort (*n* = 225), higher plasma vitamin D was associated with better scores on tests of verbal fluency and verbal memory. There was no significant association between cognitive testing and vitamin D level in the smaller (*n* = 61) demented subset (44). A second study by Gatto and colleagues assessed the role of VDR polymorphisms in cognitive decline in patients with PD (77). One hundred ninety PD patients in the Parkinson Environment Gene (PEG) study underwent the Mini-Mental State Exam (MMSE) at baseline and up to three follow-up visits and were genotyped for seven VDR polymorphisms. The FokI polymorphism was associated with decline in the MMSE score over the follow-up period after accounting for age, sex, education, and PD disease duration. Participants with AA genotype experienced faster cognitive decline as measured by change in the MMSE. A subset of study participants underwent a battery of neuropsychiatric tests, and those with the AA genotype tended to perform lower on individual neuropsychiatric tests compared to those with the AG or GG genotype (77). The VDR protein that results from the A variant has less transcriptional activity compared to the G variant allele (76), suggesting that lower VDR activity contributes to cognitive decline.

In a prospective observational study performed in England, 145 newly diagnosed PD patients were followed for 3 years and were assessed for disease severity and cognition as part of the Impairment in Cohorts with Longitudinal Evaluation—Parkinson's disease (ICICLE-PD) (50). Serum vitamin D levels were measured at baseline and 18 months. Baseline vitamin D level was not a predictor of future cognitive impairment in this cohort (50).

Several mechanisms have been proposed for the role of vitamin D deficiency in cognitive decline in the general population that may also apply to the PD population. Vitamin D may be involved in the regulation of acetylcholine and clearing of amyloid beta, both of which are implicated in the pathogenesis of cognitive impairment in PD (120, 121). Many studies demonstrate that the VDR and enzymes involved in D3 metabolism are expressed in the central nervous system, particularly in the areas of hippocampus (122, 123), and animal studies have shown that vitamin D deficiency negatively affects hippocampal learning and memory through gene expression and neural development (124). These studies suggest potential mechanisms for the effect of vitamin D on cognitive impairment in PD, but more work is needed to determine if vitamin D status affects cognition in PD and whether supplementation may help prevent or improve cognitive decline.

### Mood

The relationship between vitamin D status and depression has been inconsistent in the general population, with some studies demonstrating an increased risk of depression with lower levels of vitamin D (78, 79), while others have shown no association (80, 81). A meta-analysis of over 31,000 participants found that patients with depression had lower vitamin D levels compared to controls (82). Additionally, when comparing the lowest vitamin D category to the highest in cross-sectional studies, those in the lowest category were 30% more likely to have depression (82). Few studies, however, have examined the effect of vitamin D supplementation on mood. These include a randomized controlled trial in Norway in which 441 overweight or obese participants received either placebo, 20,000 IU or 40,000 IU of vitamin D weekly for a year (83). There was significant improvement in the Beck Depression Inventory in the two intervention arms at 1 year compared to the placebo (83). An open label study of fifty women with type II diabetes and depressive symptoms were given weekly vitamin D supplementation (50,000 IU of ergocalciferol) for 6 months, and there was a significant decrease in depression and anxiety over the study period (84).

Limited evidence exists for the association between depression and vitamin D concentration in Parkinson's disease. Peterson et al. found a correlation between plasma vitamin D levels and depression scores in 225 non-demented PD patients (44). A more recent study by Zhang et al. included 182 subjects with PD and examined the association between vitamin D levels and various motor and non-motor symptoms. They found that vitamin D level was correlated with depression and anxiety scores after adjusting for age, sex and BMI (73). More research is needed in this area to clarify the relationship between vitamin D status and depression in Parkinson's disease and to determine if supplementation is effective at improving mood.

The underlying mechanism by which vitamin D may affect mood is unknown, but several hypotheses exist based on vitamin D's effect on gene expression. The VDR is present in the cingulate cortex and hippocampus, both of which are involved in the pathophysiology of depression (25). In addition, vitamin D response elements have been detected in the promotor regions of serotonin genes, providing further evidence for a potential role of vitamin D in the development of depression (125).

### Orthostatic Hypotension

Orthostatic hypotension is defined by the American Academy of Neurology (AAN) as a reduction in systolic blood pressure by at least 20 mm Hg or diastolic blood pressure by at least 10 mm Hg within 3 min of standing (126). It can be symptomatic if patients experience symptoms of lightheadedness, dizziness, blurred vision, fatigue, nausea, etc., or asymptomatic (126, 127), and is estimated to affect 40–60% of PD patients throughout their disease course (128, 129).

The mechanism by which vitamin D may exert an effect on standing blood pressure is unclear. The cells of blood vessel walls all express the VDR and 1-alpha-hydroxylase, which may allow vitamin D involvement in modulating the vascular response when standing (85, 130). In addition, it has been postulated that vitamin D is a renin-angiotensin system regulator (RAS). RAS is implicated in blood pressure regulation and influences the sympathetic nervous system, therefore a vitamin D associated disruption in RAS could lead to dysfunction of the sympathetic system (87, 131).

Vitamin D status has been associated with orthostatic hypotension in one study involving PD patients and multiple studies involving the general population (85–90). In a study by Jang et al. 55 PD participants were divided into two groups—those with orthostatic hypotension (OH) and those without (130). The group with OH had significantly lower serum 25-hydroxyvitamin D and calcitriol levels. In addition, systolic and diastolic blood pressures, as well as OH symptom severity, were negatively correlated with serum vitamin D and calcitriol levels (130). Disease duration and disease severity were similar between the two groups, making it less likely that the OH group had lower vitamin D due to greater disease severity and more limited mobility. However, the presence of OH itself effects activity levels and is often correlated with disease severity. In a second study involving PD patients, nocturnal blood pressure changes were used as an indicator of autonomic dysfunction, and vitamin D levels were measured (132). Thirty-five PD patients were classified as “dippers” if there was a decline in mean nighttime blood pressure of >10% (normal finding), “non-dippers” if the decline in mean nighttime blood pressure was <10% (pathological) and “reverse dippers” if there was an increase in mean nighttime blood pressure (pathological). There were no significant differences in vitamin D level among the three groups, however, there were only 4 patients in the “dipper” group, so it is difficult to make firm conclusions (132).

In the general population, multiple smaller studies have shown associations between vitamin D level and orthostatic hypotension. McCarroll et al. demonstrated serum 25(OH)D levels were lower in elderly individuals with orthostatic hypotension compared to those without OH (86). Similarly, women over the age of 80 years were more likely to have diastolic orthostatic hypotension if they were vitamin D deficient (88). In the only longitudinal study that has been done examining vitamin D status and OH, it was found that baseline vitamin D deficiency was associated with an increased risk of developing orthostatic hypotension over 1 year follow-up in a group of 51 subjects from the general population (85). A meta-analysis that included 3,646 participants from five studies confirmed the findings that participants with low vitamin D had about twice the odds of having orthostatic hypotension than those within the normal range of vitamin D, and those with orthostatic hypotension were more likely to have lower vitamin D levels (87).

More recent larger studies, however, did not find a link between vitamin D status and OH. An analysis of data from 4,209 participants in The Irish Longitudinal Study on Aging (TILSA) and 2,640 participants in the Progetto Veneto Anziani (Pro.V.A.) study showed no association between vitamin D deficiency or insufficiency and orthostatic hypotension (131, 133). In a subanalysis of a randomized controlled trial assessing vitamin D supplementation for systolic hypertension, high dose supplementation did not improve orthostatic hypotension over a 12 month period (90). It is not clear why these discrepancies exist, but differences in the populations studied may have contributed. At this time, we have conflicting studies from the general population regarding the relationship between orthostatic hypotension and vitamin D, and only one study supporting the relationship in PD, so further studies are needed.

### Olfactory Function

Olfactory impairment is a common non-motor symptom in Parkinson's disease, with a prevalence of 50–90% (134–136). It is often one of the first signs of PD (137), which correlates with early involvement of the olfactory bulb with synuclein pathology (138). In a study of 39 *de novo* PD patients in South Korea, serum 25(OH)D3 level was associated with odor identification score using sniffin' sticks (91). The serum 25(OH)D3 level was also negatively correlated with subjective olfactory dysfunction. The authors postulate that vitamin D plays a role in the pathogenesis of olfactory dysfunction in PD through several potential mechanisms involving calcitriol, the active form of vitamin D (91). Calcitriol is involved in neuronal cell differentiation and neuroprotection through the VDR (6, 139, 140). A study by Glaser et al. demonstrated that the rat olfactory system contains VDR throughout (141), indicating a potential neuroprotective role for calcitriol through VDR signaling. In addition, alterations in dopamine and acetylcholine signaling have been implicated in olfactory dysfunction in Parkinson's disease (142, 143). Calcitriol has been shown to increase choline acetyltransferase and tyrosine hydroxylase activity, which are enzymes responsible for the synthesis of acetylcholine and dopamine, and may in turn exert a neuroprotective effect (144). Lastly, calcium signaling is thought to play a central role in the function of olfactory receptor neurons and can be affected by vitamin D (145). While several possible mechanisms exist for the effect of vitamin D on olfactory dysfunction in Parkinson's disease, the evidence is preliminary, and more study is needed to make any conclusions.

## Conclusions

The relationship between vitamin D status and Parkinson's disease remains unclear, and whether or not supplementation is beneficial beyond bone health in PD has yet to be determined. While preclinical studies in animals and cell culture have shown promising neuroprotective effects of vitamin D, studies in humans have been inconsistent, likely in part due to differing methodologies and populations studied. The most consistent finding in the literature is a higher prevalence of vitamin D deficiency and insufficiency in PD compared to controls, as well as an inverse relationship between vitamin D level and motor severity, but these findings may be due to reduced mobility and decreased sun exposure as PD progresses rather than disease modification by vitamin D. Much less is known about the relationship between vitamin D levels and non-motor symptoms. However, given the numerous potential benefits and limited risks, vitamin D level assessment in PD patients and supplementation for those with deficiency and insufficiency seems justified.

## Author Contributions

MF contributed to the concept of the review, performed the literature review, and drafted the manuscript. JD contributed to the concept of the review and revised the manuscript for intellectual content.

## Conflict of Interest

The authors declare that the research was conducted in the absence of any commercial or financial relationships that could be construed as a potential conflict of interest.
